# A multimodal single-cell framework for ecosystem-level profiling of circulating tumor and non-tumor cells

**DOI:** 10.1186/s40164-026-00796-y

**Published:** 2026-06-19

**Authors:** Gabriela Felix, Francesca Aguirre, Lisa Zhou, Aaron Denmark, Rocio Alvarez, Daniel M. Kim, Jun Gong, Megan P. Hitchins

**Affiliations:** 1 Department of Biomedical Sciences, Cedars-Sinai Medical Center, Los Angeles, CA USA; 2 Department of Cancer Epidemiology, Moffitt Cancer Research Center, Tampa, FL USA; 3 Department of Medicine, Cedars-Sinai Medical Center, Los Angeles, CA USA

**Keywords:** Circulating tumor cells, Single-cell multimodal profiling, Colorectal cancer

## Abstract

Multimodal profiling of circulating tumor cells (CTCs) and non-tumor circulating cells was performed in six patients with advanced colorectal cancer to identify biological programs beyond enumeration-based analyses. Across all patients, we identified 3,981 CTCs characterized by pronounced EpCAM/L1CAM co-expression, variable PD-L1 expression, and low LGR5 expression, consistent with metastatic-associated phenotypes. Integrated single-cell multi-omics analysis of 1,720 cells revealed genomic instability within EpCAM⁺, L1CAM⁺, LGR5⁺, and PD-L1⁺ CTC subsets, including Y-chromosome loss in male patients and marked intra-patient heterogeneity. In parallel, non-CTCs exhibited upregulation of inflammatory and metabolic dysregulation programs. This hypothesis-generating study highlights the relevance of blood-based multimodal single-cell profiling for interrogating tumor–host states beyond insights from CTC enumeration alone.

**Supplementary Information:**

The online version contains supplementary material available at 10.1186/s40164-026-00796-y.

## To the editor

Colorectal cancer (CRC) remains a leading cause of cancer-related mortality, driven largely by metastatic progression [1–3]. Although circulating tumor cells (CTCs) offer a minimally invasive window into metastatic disease, current approaches rely heavily on tumor-restricted markers and enumeration only, limiting biological interpretation. Given that cancer progression reflects coordinated tumor–host interactions [4, 5], we hypothesized that integrated multimodal profiling of both tumor-derived and non-tumor circulating cells could reveal system-level programs.

We profiled six patients with metastatic CRC undergoing neoadjuvant chemotherapy, including longitudinal sampling over six-months from one patient, using custom multi-marker sorting combined with comprehensive single-cell multi-omics (genome and transcriptome) sequencing to interrogate coordinated tumor–host cellular programs in peripheral blood. Detailed methods are described in the Supplementary Materials. The median age at diagnosis was 49.1 years (range, 39–71 years), and four patients were male (Supplementary Table S1).

**Fig. 1:**
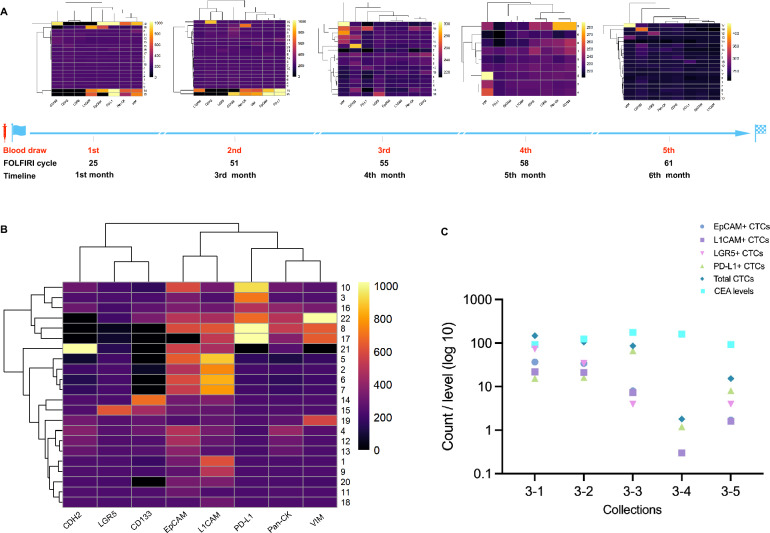
Longitudinal distribution of circulating tumor cells (CTCs) and non-CTCs isolated from the peripheral blood of Case #3 with advanced colorectal cancer.** A** Heatmaps of single-cell expression profiles organized by Phenograph clustering across five longitudinal collections spanning six months during second-line FOLFIRI treatment in stage IV colon cancer Case #3 (Q scores: 0.73–0.81). Blood samples were collected prior to each treatment cycle. **B** Phenograph clustering of 2,673 CD45⁻ cells enriched for EpCAM + and/or L1CAM + populations from Case #3 (Q = 0.91). **C** Log_10_-scaled CTC counts for individual marker-defined populations (per mL blood), total CTC counts (sum across all markers per mL), and corresponding CEA levels (ng/mL) across longitudinal collections from Case #3 (all collections, 1–5), highlighting the heterogeneity of CTC populations and limitations of single-marker enumeration. Heatmaps indicate compensated marker expression levels (yellow = highest, black = lowest) for EpCAM, L1CAM, LGR5, PD-L1, CD133, CDH2, vimentin (VIM), and pan-cytokeratin (Pan-CK)

We observed evidence that reliance on a single marker, or on tumor-restricted markers alone, may be insufficient to provide informative biological insight into overall disease burden. In the longitudinal case (#3), high-dimensional single-cell clustering analysis identified strong co-expression of EpCAM⁺ and L1CAM⁺, defining a shared metacluster with PD-L1⁺ cells, whereas LGR5⁺ and CD133⁺ cells occupied more distantly related clusters. Both marker expressions and cell counts varied over time. However, PD-L1^+^ and LGR5^+^ average counts were higher than EpCAM^+^ or L1CAM^+^, highlighting limitations of single-marker enumeration (Fig. 1).

Consistently, self-organizing map–based clustering analysis within the viable CD45⁻ cell subset from all CRC cases showed that EpCAM^+^ and L1CAM^+^ were strongly correlated and shared a metacluster with PD-L1^+^. Notably, states characterized by high PD-L1 expression were associated with reduced EpCAM or L1CAM expression. In contrast, LGR5^+^ and CD133^+^ formed a separate metacluster, consistent with the non-exclusive nature of marker relationships among CTC populations (Supplementary Fig. 1). Together, these findings, as well as the enumeration analysis in this CRC case series, highlight that single-marker enumeration is insufficient to resolve CTC heterogeneity; for example, LGR5⁺ CTC counts exceeded the EpCAM^+^ counts, a marker commonly used to identify CTCs (Supp Fig S1). In addition, CTC counts, including EpCAM⁺-only cells, showed no association with CEA levels (Supp. Fig. S2), suggesting that these markers reflect distinct biological processes, as expected. A similar inverse relationship between EpCAM⁺ cells and CEA levels has been reported in gastric cancer [6].

**Fig. 2:**
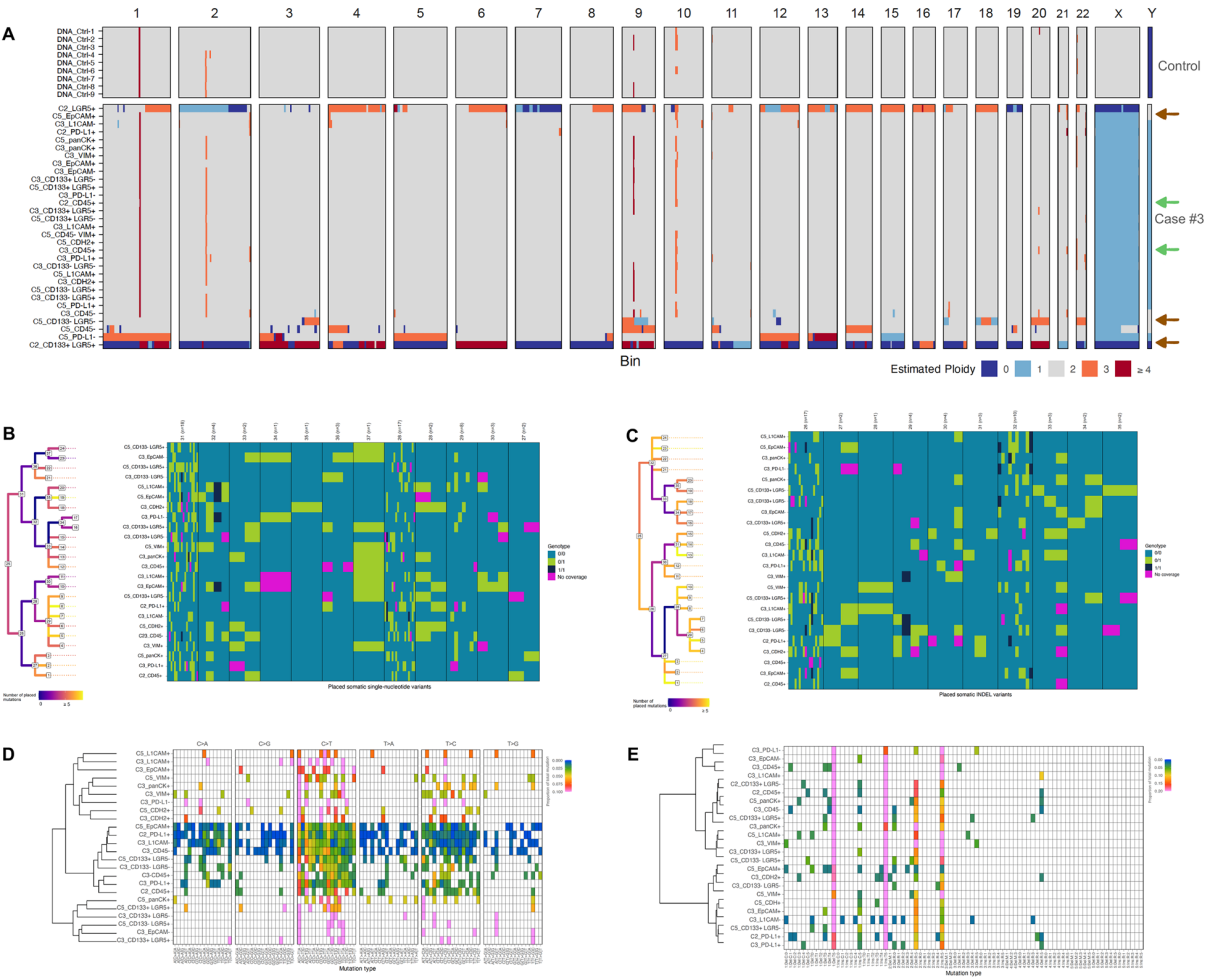
Longitudinal whole-genome amplification and mutational characterization of single cells from the peripheral blood of Case #3 with advanced colorectal cancer.** A** Genome-wide chromosomal instability (CIN) signatures across circulating tumor cells (CTCs) and non-CTCs isolated from Case #3, and the kit internal control. Subsets of EpCAM^+^, LGR5^+^, and PD-L1^−^ cells in collections C2 and C5 exhibit pronounced chromosomal instability, including copy number alterations affecting autosomes as well as sex chromosomes. These alterations include patterns of X chromosome loss with Y gain, concurrent X and Y gain, isolated Y gain, and loss of both X and Y chromosomes (brown arrows). Matched CD45^+^ cells (green arrows), as well as kit control samples, show no evidence of CIN or sex chromosome alterations. **B–C** Inferred placement of somatic mutations across CTCs and non-CTCs from Case #3, based on single-nucleotide variants (SNVs) and insertion–deletion mutations (INDELs), respectively. Branch color intensity reflects the relative number of somatic mutations, ranging from dark purple (lowest) to yellow (highest). **D**, **E** Heatmaps with associated dendrograms showing mutational burden and spectrum across CTCs and non-CTCs from Case #3, based on SNV substitution classes and INDEL categories. Tile colors represent the proportion of each mutational class per cell. For visualization purposes, the color scale is capped at 0.1 (10%), with pink indicating values ≥ 0.1. This allows representation of cells in which a single mutational class accounts for the majority of observed mutations. SNVs are broadly distributed across cells, whereas INDELs are restricted to subclonal lineages, consistent with continuous versus episodic mutational processes. Apparent copy number changes observed in chromosomes 1, 2, 9, and 10 are consistently detected across control samples and most cells, suggesting these regions reflect low coverage artifacts rather than true biological variation. Color intensity reflects estimated copy number, ranging from dark blue (0 copies) to red (≥ 4 copies)

Using integrated multi-omics single-cell sequencing, we further characterized 1720 sorted cells and detected a heterogeneous spectrum of CTCs, as shown by single-cell surface-expression data. Across EpCAM⁺, L1CAM⁺, LGR5⁺, and PD-L1⁺ subsets, we observed significant chromosomal instability (CIN), including Y-chromosome loss in male patients, which was not detected in kit controls or in matched-host CD45^+^ cells (Fig. 2A and Supplementary Fig. S3A). In bladder cancer loss of the Y chromosome has been linked to CD8⁺ T-cell exhaustion and enhanced responsiveness to anti–PD-1 therapy [7]. This underscores the utility of CTC-resolved molecular profiling in decision-making strategies.

Phylogenetic inference based on somatic mutation type also highlighted inter-sample differences, with cells clustering primarily by collection time-point (Fig. 2B, C) or case (Supplementary Fig. S4A, B), as expected. CTCs harbored more somatic mutations than matched CD45⁺ cells. In CTCs, single nucleotide variants (SNVs) were broadly distributed and contributed predominantly to early branches, whereas indels were enriched in later branches. Notably, indels were concentrated in subclonal stem-like (e.g., CD133^+^/LGR5⁺ or LGR5^−^ subsets), L1CAM⁺, and mesenchymal CTC populations, and were largely absent from epithelial EpCAM⁺ CTCs and CD45⁺ cells (Fig. 2B, C; Supplementary Fig. S4A, B). Similar lineage-specific indel patterns have been reported in other tumor types, including lung, gastric, and thyroid cancers [5]. In our case series, lung was the most common metastatic site (Supplementary Table S2), suggesting a potential link between these evolutionary patterns and metastatic progression, although larger studies are needed to confirm this association.

Within the longitudinal CRC case, with lung metastasis, the LGR5^+^ and L1CAM^+^ CTCs harbored the highest somatic SNV and indel burdens, respectively (Fig. 2B, C). Across all cases, after PD-L1^−^, LGR5^+^ CTCs also harbored more somatic SNVs, whereas LGR5^−^ CTCs harbored more indels (Supplementary Fig. S4A, B). Beyond highlighting the mutational signature patterns of CTCs, these findings underscore the potential utility of LGR5 in the metastatic CRC context, particularly in the LGR5^−^ state that has been shown to promote CRC metastasis in preclinical studies [8].

The single-cell transcriptomic landscape further revealed evidence of tumor–host interactions. Within the host-compartment, matched CD45^+^ cells exhibited upregulation of immune signaling and T-cell activation transcripts (e.g*., RHBDF2, SLAMF6, AZI2, CASP8AP2*), whereas matched CD45⁻ populations were enriched for genes associated with tumor growth, inflammatory, and metabolic programs (e.g., *S100A8/A9, GPR171, CD27, MYL6*)*,* as shown in Supplementary Fig. S5C. Half of the cases exhibited low BMI prior and at the time of blood collection, suggesting possible cancer-associated cachexia (Supplementary Fig. S6). However, given the limitations of BMI as a measure, further evaluation of metabolic markers in the context of cancer is warranted.

Notably, inclusion of CD45⁻/PD-L1⁺ cells in the transcriptome analysis revealed upregulation of NK-associated genes, indicating immune-like transcriptional features and limiting the utility of PD-L1 alone for CTC detection. Consistent with this observation, PD-L1 expression has been reported in both tumor and immune cells and is constitutively expressed in certain non-lymphoid tissues, including the heart and lung [9–12], underscoring the need for contextual interpretation.

Within the tumor compartment, across CTC subsets, markers associated with immune-promoting functions (e.g., *APOL3, LBH,* and *IL7*) were downregulated, whereas *IER2*—a gene associated with metastasis and poor prognosis—was upregulated, consistent with the tumor–host interactions at the tumor–blood interface in CRC (Supplementary Fig. S5D). The CTC subsets also exhibited distinct CRC driver expression profiles (Supplementary Fig. S5E) and were enriched for pathways associated with epithelial–mesenchymal transition, immune–stromal interactions, and metabolic programs, supporting their tumor origin (Supplementary Fig. S7).

Consistent with these findings, prior studies have reported transcriptomic heterogeneity and pathway variability in CTCs and immune cells across tumor types and anatomical compartments, although these have typically been analyzed separately [13, 14]. In our study, application of a multimodal liquid biopsy framework enabled simultaneous interrogation of tumor and host compartments at single-cell resolution, revealing tumor-intrinsic mutational signatures alongside host immune and metabolic perturbations in non-CTCs from advanced CRC. Further studies in larger and more diverse cohorts—including control individuals with non-malignant gastrointestinal inflammatory conditions (e.g., Crohn’s disease or diverticulitis)—are warranted to better define the cellular states underlying tumor–host interactions and their role in metastatic evolutionary processes in CRC.

## Supplementary Information

Supplementary Material 1.

Supplementary Material 2.

Supplementary Material 3.

Supplementary Material 4.

Supplementary Material 5.

Supplementary Material 6.

Supplementary Material 7.

Supplementary Material 8.

Supplementary Material 9.

Supplementary Material 10.

Supplementary Material 11.

## Data Availability

Availability of data and materials: Analyses were performed using publicly available bioinformatics software with documented workflows and cited versions, following standard command-line usage as described in the Supplemental Methods. No custom algorithms were developed. De-identified data generated in this study are available from the corresponding authors upon reasonable request.

## Code availability

No custom code was generated in this study.
