# Supplementary material for: A multimodal single-cell framework for ecosystem-level profiling of circulating tumor and non-tumor cells
Source: Exp Hematol Oncol. 2026 Jun 19;15:54. doi: 10.1186/s40164-026-00796-y (PMC13281560; doi:10.1186/s40164-026-00796-y)
Supplement: Supplementary file 1 — Supplementary Material 1. [file 40164_2026_796_MOESM1_ESM.docx]

**A Multimodal Single‑Cell Framework for Ecosystem‑Level Profiling of Circulating Tumor and Non‑Tumor Cells**

**Supplemental Material**

**Methods**

**Human Subjects**

Between May and December 2024, six patients with advanced colorectal cancer (AJCC stage III or IV) were prospectively recruited at the Cedars‑Sinai Samuel Oschin Comprehensive Cancer Institute (Los Angeles, CA, USA), without restrictions on age, sex, or race/ethnicity. All participants were unrelated individuals who were undergoing systemic treatment at the time of enrollment, and blood samples were collected prior to their most recent systemic treatment. In addition, two cancer‑free individuals were included as controls. Sex and race/ethnicity were self‑reported, and sociodemographic data were collected at enrollment using a standardized questionnaire administered by a trained research coordinator. Written informed consent was obtained from all participants. The study was approved by the Cedars‑Sinai Institutional Review Board (IRB #Pro00054104) and conducted in accordance with institutional and national ethical standards and the 1964 Declaration of Helsinki. None of the control participants or CRC cases had a documented clinical history of inflammatory gastrointestinal conditions (e.g., Crohn’s disease or diverticulitis) or HIV at enrollment, and both groups remained free of these conditions during two years of follow-up.

**Sampling and Clinical Data Collection**

Blood samples were collected from each of five colorectal cancer (CRC) patients prior to the start of a new treatment cycle during ongoing systemic therapy (Supplementary Table S1). For serial validation and temporal analysis, metastatic CRC Case #3 was sampled five times, once prior to the initiation of a new cycle of neoadjuvant chemotherapy, and an additional four times thereafter over a six‑month period while the patient continued to undergo neoadjuvant chemotherapy. One monthly time-point was missed because the participant did not attend the scheduled appointment. Two healthy control subjects were included, both mSEPT9 negative and one colonoscopy-verified, who remained cancer-free for two-years of follow-up (Supplementary Table S2).

Peripheral blood samples (10–20 mL) were collected using BD Vacutainer® K2EDTA tubes or Streck Cell‑Free DNA BCT™ tubes and processed within one hour of collection. All blood draws were performed via standard venipuncture by a certified phlebotomist. The total volume of blood obtained from each patient ranged from 10 mL to 20 mL, depending on venous access.

Only clinicopathologic data relevant to the study objectives were extracted from medical records by the clinical research coordinator. Researchers involved in patient recruitment did not participate in laboratory procedures or data analysis, and these personnel were blinded to the recruitment process.

**Single-Cell Enrichment Protocols**

Peripheral blood samples underwent single-cell enrichment using either Single-Cell Cell Select Slides™ v1 with the Genesis system (BIO-RAD, USA) following manufacturer instructions or a validated in-house protocol. The in-house method involved centrifugation at 1,200 × g for 12 min, removal of two-thirds of the plasma, and subsequent red blood cell (RBC) lysis of the remaining sample using eBioscience™ 1× RBC Lysis Buffer (Invitrogen™, Thermo Fisher Scientific) to deplete erythrocytes. After washing in nuclease-free PBS, centrifugation (400 × g for 8 minutes), and resuspension of intact cells in nuclease‑free PBS containing 0.02% UltraPure™ BSA, the cells were divided into three tubes designated for: (A) no markers, (B) CD45 only, and (C) all markers (CD45, EpCAM, PD‑L1, VIM, L1CAM, CDH2, LGR5, CD133, and pan‑cytokeratin). Details for each marker are provided in Supplementary Table S1.

Ghost Dye™ Violet 510 (CYTEK®) was added for viability assessment in all three tubes, followed by Fc receptor blocking (BD Pharmingen™ Human BD Fc Block™) in tubes B and C. Tube B was then stained with CD45 only; tube C underwent multiplex staining using monoclonal antibodies listed in the Supplementary Table S1 including the CD45 marker. Cells were washed, resuspended in BD Pharmingen™ Stain Buffer, and processed immediately by flow cytometry using the BD FACSAria™ III. Sorted cells were deposited into 96-well plates preloaded with ResolveOME Cell Buffer (BioSkryb Genomics), sealed, and stored at −80 °C until next-generation sequencing (NGS) library preparation.

**Fluorescence-Activated Cell Sorting (FACS) Data Analysis**

FACS data for all cell-surface markers (see Supplementary Table S3) were analyzed using FlowJo™ v10. Cells were initially gated for viability (GhostDye) and CD45 expression. Viable CD45⁻ single cells (which also include CTCs) were randomly selected and further assessed for individual or combinatorial expression of eight markers: EpCAM, PD-L1, VIM, L1CAM, CDH2, LGR5, CD133, and pan-cytokeratin. Cells were classified as CTCs if they were CD45⁻ and expressed EpCAM, L1CAM, or both. These cells were further evaluated for expression of additional markers, including PD-L1, VIM, CDH2, LGR5, CD133, and pan-cytokeratin. For normalization, data from viable CD45⁻ cells from each collection from Case #3 were randomly selected and concatenated into a dataset comprising 17,241 CD45⁻ single cells. From this concatenated dataset, along with the datasets from the five additional samples from the other CRC cases, data from approximately 5,000 cells were randomly selected from each sample, yielding a final dataset of 32,241 CD45⁻ single cells. Finally, from this pooled dataset of 32,241 CD45⁻ single cells, we constrained the random selection to cells primarily expressing EpCAM⁺ and/or L1CAM⁺, yielding a total of 3,981 single cells. These were used to evaluate the dynamics of expression of the remaining markers (PD-L1, VIM, CDH2, LGR5, CD133, or pan-cytokeratin) within this subset.

In viable CD45⁻ cells from control subjects, VIM and CD133 were the only markers significantly expressed, consistent with progenitor cell profiles in peripheral blood within the healthy context. High-dimensional clustering and visualization were performed using UMAP, t-SNE, and Self-Organizing Maps (SOMs), implemented in FlowSOM v4.1.0 [1], EmbedSOM v2.2.0 [2], and Phenograph v4.0.5 [3] in RStudio (Version 2025.05.0+496; R v4.4.3), using default parameters for FlowJo™ v10.8 Software (BD Life Sciences) as previously described.

**Molecular Characterization of Sorted Peripheral Nucleated Cells through Single-Cell Sequencing**

Frozen 96-well plates containing patient-specific sorted cells sharing cell-surface marker profiles were thawed on ice and centrifuged prior to library preparation. Each well contained 7–10 cells sorted by common surface markers and patient of origin. Matched CD45⁺ and CD45⁻ cells served as controls, representing paired basal normal tissue. Including these matched controls, a total of 184 libraries were generated from ~1,840 single cells isolated from the six advanced CRC cases. In addition, BioSkryb ResolveOME quality controls for both RNA and DNA were performed in triplicate across three independent experiments, along with negative controls containing buffer only.

Paired-end single-cell libraries were prepared using the ResolveOME™ Whole Genome and Transcriptome Single-Cell Core Kit v2 (BioSkryb Genomics, USA) with unique dual indexes (UDI), according to the manufacturer’s instructions. Library quality was assessed using the Qubit™ 1X dsDNA High Sensitivity Assay (Thermo Fisher Scientific) and the Femto Ultra Sensitivity NGS Kit on the Femto Pulse System (Agilent Technologies). Femto Pulse analysis showed that libraries from single cells and controls exhibited fragment size distributions ranging from 200 to 800 bp, while no amplified fragments were detected in negative controls.

A total of 172 single-cell libraries, including 154 qualified libraries and 18 DNA/RNA commercial controls provided with the kit, were sequenced on the Illumina NovaSeq 6000 platform (PE100 flow cell). Whole-genome amplification (WGA) libraries targeted approximately 20× coverage (10 million paired-end reads per library), while RNA libraries targeted 100 million paired-end reads. Sequencing was performed at the Cedars-Sinai Applied Genomics, Computation & Translational Core (Los Angeles, CA, USA).

**Single-cell Whole-Genome Amplification (WGA) Data Analysis**

Single-cell WGA FASTQ files were processed using the BJ-Germline-Variant calling-Parabricks pipeline. Reads were aligned to GRCh38 and duplicates removed with Parabricks FQ2BAM. Variant calling employed Google DeepVariant, trained on BioSkryb single-cell data, incorporating population allele frequencies for improved sensitivity. Alignment and coverage metrics were assessed using Parabricks COLLECTMETRICS.

Somatic variant calling used a two-step heuristic filtering strategy as follows: First, Binomial and Beta-binomial Filtering – Aggregated read counts across cells were tested using binomial and beta-binomial models (parallelized by chromosome) to remove germline variants and low-input artifacts (Sequoia framework). Depth filters were applied based on mean coverage. Secondly, quality Control Filtering – BAM pileups were analyzed for strand-specific support, alignment scores, clipping, and positional statistics (SD, MAD). Per-cell thresholds included alignment score ≥140, clipped reads ≤25%, and positional bias. Group-level filters required ≥3 high-quality reads per cell and ≥70% of cells with sufficient coverage.

Variants passing all filters were used to construct phylogenetic matrices. A second Sequoia filtering round preceded phylogenetic inference using MPBoot, with somatic variants mapped to inferred lineages via Treemut. Visualization employed ohchibi and ggtree in R. Mutational profiles were generated using SigProfiler MatrixGenerator; SBS96 contexts were visualized in ggplot2. Genomic feature intersections utilized VariantAnnotation, TxDb.Hsapiens.UCSC.hg38.knownGene, BSgenome.Hsapiens.UCSC.hg38, and org.Hs.eg.db.

**DNA copy number variation (CNV) Inference and Quality Control**

The same aligned, duplicate‑removed BAM files generated for somatic variant calling were used for CNV inference and DNA quality assessment using the BaseJumper™ DNA workflow. Read depth was aggregated into genomic bins across each chromosome, and absolute ploidy was inferred from normalized read‑depth ratios and discretized into integer copy‑number states (0, 1, 2, 3, or ≥4), which were visualized as genome‑wide ploidy heatmaps.

Per‑cell DNA quality was assessed using standard BaseJumper™ DNA QC metrics, including MAPD (Median Absolute Pairwise Difference) of CNV, unevenness of CNV, percentage of genome covered at ≥5× depth (PCT5× coverage), proportion of chimeric reads, and proportion of mitochondrial read mapping. MAPD of CNV was used as the primary indicator of amplification noise introduced during whole‑genome amplification. Cells failing prespecified DNA QC thresholds were designated low pass and excluded from CNV interpretation, whereas high‑pass cells were retained.

Across patients, the majority of cells exhibited greater than 30% genome coverage at ≥5× depth, with only a small subset (8 of 155 cells) showing reduced PCT5× coverage of approximately 15%. To assess the robustness of downstream analyses to inclusion of these lower‑coverage cells, the Somatic Heuristic Filtering (SHF) pipeline was executed twice for Case #3 (serial collection) — once including and once excluding these cells. Comparison of the resulting phylogenetic trees showed minimal differences in topology (p < 2.2 × 10⁻¹⁶), indicating that inclusion of the lower‑coverage cells did not meaningfully affect inference. Based on this robustness analysis, these cells were retained for downstream analyses.

CNV‑specific quality assessment across the dataset demonstrated that 96% of cells fell within the ideal quality zone, defined by low MAPD of CNV and low unevenness of CNV, corresponding to a maximum MADPD threshold of 0.2, supporting high confidence in the inferred CNV profiles.

Sex chromosomes were evaluated using absolute copy‑number estimates, consistent with BaseJumper™ CNV reporting. As expected, sex chromosomes—particularly chromosome Y—showed modestly elevated MAPD values due to haploid baseline copy number, reduced mappability, repeat‑rich sequence content, and increased sensitivity to amplification variance in smaller chromosomes. Accordingly, sex‑chromosome CNVs were interpreted based on consistency of inferred ploidy states across cells and concordance with autosomal CNV structure, rather than MAPD values alone. Estimated ploidy values reflect absolute copy number; therefore, ploidy = 2 on chromosome Y represents gain relative to the normal male haploid state.

**Single‑Cell Whole RNA Data Analysis**

Single‑cell RNA‑sequencing data were processed using the BJ‑Expression pipeline. Raw FASTQ files underwent sequencing quality assessment, adapter trimming, and filtering using FASTP and Cutadapt, followed by read down‑sampling. Transcript‑level quantification was performed using Salmon, and splice‑aware alignment was conducted with STAR. Gene‑level counts were generated using HTSeq, and alignment quality metrics were evaluated with Qualimap. Quality metrics were aggregated across samples using MultiQC.

**RNA Quality Control and Filtering**

Per‑cell RNA quality assessment was performed using custom RNA QC metrics implemented in the BJ‑Expression workflow, including proportion of intergenic reads (Prop.intergenic < 0.2, proxy for DNA contamination), proportion of mitochondrial reads (Prop.mitochondrion < 0.2), and number of detected protein‑coding genes (>1,000). Cells passing RNA QC were retained for downstream analyses.

**RNA Normalization and Downstream Analyses**

Filtered gene‑expression matrices were generated using a logarithmic normalization approach (counts per cell divided by total counts, scaled, and log₂‑transformed). These matrices were used for Constrained Analysis of Principal Components (CAP) via the oh.cap function in the ohchibi R package and for differential gene expression analysis using zero‑inflated linear models (ZLM) implemented in MAST. Volcano plots were generated using ggplot2.

Standardized expression matrices were also used for gene set enrichment analysis (GSEA) and for additional differential expression and pathway analyses using iDEP v2.4.3 / iDEP96. Cell phenotypes were defined based on cell‑surface marker profiles. Overall RNA‑sequencing quality assessment indicated that approximately 70% of cells (55 of 75 cells across 10 collections), excluding external controls, passed RNA QC and were retained.

**Plasma mSEPT9 testing**

Plasma removed following the first centrifugation of each peripheral blood sample was centrifuged again at 10,000 x g and frozen at -80°C. As we have previously described, mSEPT9 ctDNA analyses were performed on 1 mL volumes of plasma using the Epi proColon 2.0 CE kit (Epigenomics Inc.) with modifications, and levels measured as percentage of methylated reference (PMR) values[4,5].

**Data Analysis**

Non-parametric correlation analyses of clinical, AST/ALT, CEA, mSEPT9 levels and CTCs data were performed using GraphPad Prism v10.4.2 with α = 0.05 and 95% CI.

**Supplementary Figure S1. Expression and count dynamics of circulating tumor cells (CTCs) from the peripheral blood of patients with advanced colorectal cancer.**

**(A)** FlowSOM self-organizing map (SOM) heatmap showing marker expression dynamics across 3,981 randomly selected EpCAM+ and/or L1CAM+ CTCs from all cases.

**(B)** Log₁₀-scaled CTC counts for individual marker-defined populations (per mL blood), total CTC counts per mL (sum across all markers), and corresponding CEA levels (ng/mL) across all colorectal cancer cases, highlighting the heterogeneity of CTC populations and limitations of single-marker enumeration.

**(C-D)** FlowSOM t-SNE meta-cluster distribution and corresponding cell-surface marker expression patterns in EpCAM+ and/or L1CAM+ CTCs.

**(E)** Minimum spanning tree (MST) representation of CTC clusters, illustrating topological relationships and associated marker expression profiles.

*Note: Heatmaps indicate compensated marker expression levels (yellow = highest, black = lowest) for EpCAM, L1CAM, LGR5, PD-L1, CD133, CDH2, vimentin (VIM), and pan-cytokeratin (Pan-CK).*

**Supplementary Figure S2. Non‑parametric correlation matrix of clinical features and standard‑of‑care markers versus circulating tumor cell (CTC) markers in the advanced CRC case series.**

Non-parametric correlation analysis illustrates relationships among age at diagnosis, BMI, CEA, mSEPT9, AST, ALT, and CTC counts (including panel (CD45⁻/EpCAM^+^/L1CAM^+^/PD-L1^+^/LGR5^+^) and subsets) and CD45⁻/EpCAM^+^ only subset). Spearman coefficients are displayed within clusters, with strong positive correlations highlighted in yellow and strong negative correlations in black, revealing key associations between circulating tumor burden and clinical biomarkers.

**Supplementary Figure S3. Genome-wide chromosomal instability (CIN) patterns in single cells from advanced colorectal cancer cases.**

Genome-wide copy number profiles derived from whole-genome amplification across circulating tumor cells (CTCs) and non-CTCs from advanced colorectal cancer cases (#1–#6), and kit internal controls. .

A subset of EpCAM^+^, L1CAM^+^, LGR5^+^, PD-L1^+^ and PD-L1^-^ cell populations from Cases #1, #2, and #3 exhibit pronounced chromosomal instability, including copy number alterations affecting autosomes as well as sex chromosomes. These alterations include patterns of X chromosome loss with Y gain, concurrent X and Y gain, isolated Y gain, and loss of both X and Y chromosomes (brown arrows).

In contrast, matched CD45+ cells from all cases (green arrows and lines), as well as kit control samples, show no evidence of CIN or sex chromosome alterations. These results indicate that CIN is a consistent and distinguishing feature of tumor-derived CTC populations across patients. Male cases #1 and #3. Female cases #2, #4, #5 and #6.

*Note: The apparent copy number alteration observed in chromosomes 1, 2, 9, and 10 consistently observed across control samples and most cells, suggesting these regions likely reflect low coverage artifacts rather than true biological variation. Color intensity reflects estimated copy number, ranging from dark blue (0 copies) to red (≥4 copies).*

**Supplementary Figure S4. Inferred evolutionary placement of somatic mutations and mutational burden across circulating tumor cells (CTCs) and non-CTCs from advanced colorectal cancer patients.**

**(A–B)** Inferred evolutionary placement of somatic mutations across CTCs and non-CTCs from all advanced colorectal cancer patients, based on single-nucleotide variants (SNVs) and insertion–deletion mutations (INDELs), respectively. Branch color intensity reflects the relative number of somatic mutations, ranging from dark purple (lowest) to yellow (highest).

**(C–D)** Heatmaps with associated dendrograms showing mutational burden and spectrum across CTCs and non-CTCs from all patients, based on SNV substitution classes and INDEL categories. Tile colors represent the proportion of each mutational class per cell. For visualization purposes, the color scale is capped at 0.1 (10%), with pink indicating values ≥0.1. This allows representation of cells in which a single mutational class accounts for the majority of observed mutations (e.g., up to 100% in 259 cells).

**Supplementary Figure S5. Transcriptomic profiles of circulating tumor cells (CTCs) and non-CTCs isolated from the peripheral blood of patients with advanced colorectal cancer.**

**(A)** Distribution of CTCs and non-CTCs based on transcriptomic profiles and cell-surface marker expression **(B)** Cell distribution according to sample origin (individual patients).

**(C)** Volcano plot showing differential gene expression between CD45⁺ and CD45⁻ cell populations (log₂ fold change vs. adjusted p-value).

**(D)** Volcano plot showing differential gene expression between EpCAM⁺/L1CAM⁺/LGR5⁺ CTCs and CD45⁺ cells (log₂ fold change vs. adjusted p-value).

**(E)** Expression distribution of colorectal cancer–associated genes across all CTCs. Each point represents the per-cell z-scored expression of an individual gene, centered on the All-CTCs baseline. Black points indicate individual CTC measurements, and the thin red vertical line marks the median expression across cells.

**(F**) Hierarchical clustering based on Pearson correlation of the top 100 normalized genes across CD45+ cells, CTCs expressing EpCAM+, L1CAM+, LGR5+, or PD-L1+, and other CD45⁻ populations (maximum z-score = 3).

These results demonstrate that transcriptional heterogeneity reflects phenotypic diversity and distinct cellular states across CTC populations.

**Supplementary Figure S6. Longitudinal BMI measurements relative to liquid biopsy (LB) collection (month 0) across patients with advanced colorectal cancer.**

**(A)** Individual patient BMI measurements plotted over time (months) relative to liquid biopsy collection (month 0), spanning six months before and after sampling. Colored points represent BMI values for each subject. The solid red line indicates the group mean with shaded 95% confidence intervals, and the solid blue line represents the predicted mean trajectory estimated using a simple linear model. A modest decline in BMI is observed in the months preceding and following LB collection.

**(B)** Heatmap of monthly changes in BMI (ΔBMI, kg/m²) relative to each patient’s BMI at the time of LB collection. Positive values (red) indicate increases in BMI, whereas negative values (blue) indicate decreases. While patterns vary across patients, several individuals exhibit reductions in BMI prior to LB collection, consistent with the trend observed in panel A.

**Supplementary Figure S7. Functional analysis using Gene Set Enrichment Analysis (GSEA) showing plots of pathways positively correlated in circulating tumor cells (CTCs) expressing EpCAM+, L1CAM+, LGR5+, or PD-L1+ compared with CD45+ cells classified as normal.** *Sample name colors: gray = CTC; yellow = CD45⁺.*

**References**

1. Van Gassen S, Callebaut B, Van Helden MJ, Lambrecht BN, Demeester P, Dhaene T, et al. FlowSOM: Using self-organizing maps for visualization and interpretation of cytometry data. Cytometry Part A [Internet]. Wiley-Liss Inc.; 2015 [cited 2026 May 11];87:636–45. https://doi.org/10.1002/CYTO.A.22625;WGROUP:STRING:PUBLICATION

2. Kratochvíl M, Koladiya A, Vondrášek J. Generalized EmbedSOM on quadtree-structured self-organizing maps. F1000Research 2020 8:2120 [Internet]. F1000 Research Limited; 2020 [cited 2026 May 11];8:2120. https://doi.org/10.12688/f1000research.21642.2

3. Levine JH, Simonds EF, Bendall SC, Davis KL, Amir EAD, Tadmor MD, et al. Data-Driven Phenotypic Dissection of AML Reveals Progenitor-like Cells that Correlate with Prognosis. Cell [Internet]. Cell Press; 2015 [cited 2026 May 11];162:184–97. https://doi.org/10.1016/j.cell.2015.05.047

4. Hitchins MP, Vogelaar IP, Brennan K, Haraldsdottir S, Zhou N, Martin B, et al. Methylated SEPTIN9 plasma test for colorectal cancer detection may be applicable to Lynch syndrome. BMJ Open Gastroenterol [Internet]. BMJ Publishing Group; 2019 [cited 2026 May 25];6:e000299. https://doi.org/10.1136/BMJGAST-2019-000299

5. Loomans-Kropp HA, Song Y, Gala M, Parikh AR, Van Seventer EE, Alvarez R, et al. Methylated Septin9 (m SEPT9): A promising blood-based biomarker for the detection and screening of early-onset colorectal cancer. Cancer research communications [Internet]. Cancer Res Commun; 2022 [cited 2026 May 25];2:90–8. https://doi.org/10.1158/2767-9764.CRC-21-0142
